# An Innovating Tool to Enhance the Oral Health of Older Adults With or Without Dementia Through the 4Ms Model

**DOI:** 10.1093/geroni/igaa057.031

**Published:** 2020-12-16

**Authors:** Maryam Tabrizi, Margo Melchor

**Affiliations:** The University of Texas School of Dentistry at Houston, Houston, Texas, United States

## Abstract

Oral health of the elderly is the most neglected area of healthcare by many providers across healthcare disciplines. Often due to lack of Medicare funding and affordability of the elderly that leads to a constant high cost of non-traumatic dental related emergency department visits. By 2030, the number of adults in the USA will increase to 74 million. Over 22 million (30%) of older Americans will need specialized geriatric care. Simultaneously, in 2025, a national shortage of medical geriatricians will be close to 27,000 full time positions. This shortage has a greater impact on oral health often due to limited access, affordability and shortage of trained dentists to manage patients with special needs, particularly those with cognitive declines. There is an urgent need for an innovation for workforce enhancement nationwide. Innovation that can create a network of age-friendly dental clinics could be based on the 4Ms model-Mentation, Mobility, Medication, and Matters most. Project ECHO is a telementoring guided practice model that revolutionizes health education and increases workforce to provide best-practice specialty care by reducing health disparities. By utilization of the Project ECHO among community dentists, this makes transformation richly effective. Training oral health providers for utilizing the project ECHO model is a logical response for the shortage and to increase oral health access. Project ECHO trains general dentists & dental hygienists to provide specialty care services. Meaning the elderly can obtain the care they need in the right place, at the right time, with better treatment outcomes and reduces costs.

